# Natural Products and/or Isolated Compounds on Wound Healing

**DOI:** 10.1155/2019/4594965

**Published:** 2019-05-02

**Authors:** Christian Agyare, Abidemi J. Akindele, Vanessa Steenkamp

**Affiliations:** ^1^Kwame Nkrumah University of Science and Technology, Kumasi, Ghana; ^2^University of Lagos, Lagos, Nigeria; ^3^University of Pretoria, Arcadia, South Africa

The effective treatment of wounds remains a major global health challenge. Failure to heal or elongation of the wound healing process results in increased financial and social stress being placed on health institutions, care-givers, patients, and their families. The occurrence of various forms of wounds such as chronic and acute wounds, pressure ulcers, venous stasis ulcers, and diabetic ulcers has increased over the years in most countries, especially developed countries where life expectancy has been increasing over time and is accompanied by geriatric diseases. Acute and chronic, nonhealing wounds impose heavy financial and quality of life burdens on patients [1]. Chronic wounds are normally characterized by intense pain, infection, loss of function, and loss of mobility and may lead to amputations and in some cases even death. With an increase in the prevalence of wounds and the high cost of orthodox medicines, most patients, especially those in developing countries, resort to herbal preparations or remedies which are believed to be readily available and cheap for the treatment thereof. The urgent need for the identification of effective, safe, and cost efficient wound healing promoters which can be introduced into clinical practice is unequivocal [2, 3]. This has driven an increase in the search for potent, cost effective wound healing agents from natural products including medicinal plants.

Medicinal plants have been used in the management of various diseases since ancient time. It is estimated that about 70 to 80% of the world's population depends on medicines of plant origin for treatment of diseases. The use of medicinal plants in the management of acute and chronic wounds is common in most traditional medicine practices in the world. Based on this, many plants in the tropical and subtropical regions of the world have been screened for their wound healing activity. Yet there are still many medicinal plants that need to be screened in the search for newer, efficacious, and cost effective wound healing agents. This special issue provides the platform for bringing to the limelight recent efforts in this regards.

This issue contains eight articles which focus on studies on the current trends for managing wounds and associated complications, newer natural products including animal products, and/or isolated compounds possessing wound healing activity.

R. Komakech et al. presented a review on the wound healing potential of* Aspilia africana *(Asteraceae), a plant used in African traditional medicine to treat wounds. The authors reported that* in vitro *and* in vivo *investigations provided evidence of the wound healing properties of the plant's derived extracts and phytochemicals, including alkaloids, saponins, tannins, flavonoids, phenols, terpenoids, *β*-caryophyllene, germacrene D, *α*-pinene, carene, phytol, and linolenic acid. These phytoconstituents were linked with strong anti-inflammatory, antimicrobial, and antioxidant activity, all essential for wound healing. Specific activities of the extracts of* A. africana *and its constituents beneficial to wound healing were reported to include inhibitory effects on bleeding, enhancement of wound contraction, increases in the levels of basic fibroblast growth factor (BFGF) and platelet derived growth factor, and stimulation of haematological parameters like white and red blood cells.

M. Gulumian et al. reported on African herbal remedies with antioxidant activity as potential resource base for wound treatment in a form of a review article. This is based on the premises that excess free radicals have been linked with wound chronicity and antioxidant therapy facilitates wound healing. The review highlighted tests that have been used to assess antioxidant activities of African medicinal plant extracts; compounds isolated from African medicinal plant extracts with confirmed antioxidant activities; and crude extracts of African medicinal plants with confirmed antioxidant activities. The authors reported that either single assays or* in vitro *analysis were used to determine the antioxidant activity of listed extracts and compounds, warranting the use of alternative testing methods, including* in vivo *assays, for confirmation of observed effects. They also clamoured for identification of compounds responsible for the antioxidant activities and evaluation of wound healing properties of isolated compounds in applicable cases.

J. E. T. do Nascimento identified and isolated the main chemical compounds present in extracts of* Ouratea fieldingiana *(Ochnaceae) and investigated their possible antifungal, antioxidant, and anticholinesterase activities. The plant is popularly used in Brazilian folk medicine for wound healing and treatment of inflammation and infectious diseases. Amentoflavone and kaempferol 3-O-rutinoside were isolated, respectively, from the ethanol seeds and leaves extracts of the plant. The extracts and compounds were shown to possess antifungal activity against several* Candida *strains via inhibition of ergosterol biosynthesis, antioxidant, and anticholinesterase activities.

K. Xu et al. in a review article on plant-derived products for treatment of vascular intima hyperplasia (IH) described the different originating cells involved in vascular IH and highlighted the effect of different natural products on inhibiting abnormal cellular functions, such as vascular smooth muscle cells (VSMC) proliferation and migration. The review article covered diverse cells involved in vascular IH; antiproliferation, migration, and cellular functions of abnormal VSMCS as a target to decrease intimal hyperplasia; typical signal pathways involved in the growth and physiology of VSMCs in IH disease; different natural compounds being used for preventing neointimal formation; and selective inhibition of VSMCs versus vascular endothelial cells (VECs).

In another review article, L. Tan et al. evaluated the clinical effective rate, safety, and financial cost of traditional Chinese medicine injections (TCMIs) in treating diabetic foot and ulcer wound healing. The findings from the study suggest that TCMIs are beneficial to patients with diabetic foot ulcers, increasing the clinical effective rate of conventional therapies, and eliciting better performance in safety and financial burden. The authors however suggested more rigorous designed randomized control trials (RCTs) with large sample size to provide more high-quality evidence to support the benefits of TCMIs in the treatment of various types of wounds.

K. Jenwitheesuk et al. evaluated the efficacy of* Centella asiatica *(Apiaceae) extract in cream for the prevention of scar development of the split-thickness skin graft (STSG) donor site. A prospective randomized, controlled, double-blind trial was conducted on the* Centella *cream for scar improvement. The amelioration of hypertrophic scar by the extract cream was attributed to better pigmentation, improvement of objective measurements, and longer follow-up times.

M. Çalışır et al. investigated the effect of humic acid on the healing of excisional wounds in the palate of rats as a follow-up to previous demonstration of enhancement of cutaneous wound healing and antibacterial properties. The findings from the study showed that humic acid treatment enhanced the rate of wound closure and recovery. This provides a basis for the use of humic acid as an alternative in the treatment of oral wounds.

In the last publication in this special issue, J. Kim et al. in a research article investigated the effect of tracheloside, a plant lignin, on keratinocyte proliferation using scratch wound healing and cell proliferation assays and western blot analysis based on the fact that cell migration and proliferation are important for proper wound healing after skin injury. In the study, it was demonstrated that tracheloside positively affects the proliferation of HaCaT keratinocyte cell line through the regulation of ERK1/2 phosphorylation. The authors concluded that tracheloside is a potential therapeutic candidate for promotion of wound healing.

The high quality articles constitute authors located at affiliations and institutions from nine different countries and parts of the world. This special issue has extended the frontiers of knowledge in exploring the therapeutic application of natural products and/or isolated compounds in wound healing which accounts for significant morbidity and mortality worldwide. It has also provided the desired basis for future research in complementing and/or replacing existing therapeutic agents used in the treatment of wounds.
